# Crossing the Innovation Chasm: Identifying Facilitators and Barriers to Early Adoption of the Global Health Starter Kit Curriculum

**DOI:** 10.5334/aogh.3356

**Published:** 2021-11-22

**Authors:** Jennifer Lee, Ethan Tan, Jane Barrow, Candace Bocala, Brittany Seymour

**Affiliations:** 1 Harvard School of Dental Medicine (HSDM), US; 2 Fourth year HSDM student, US; 3 HSDM Initiative to Integrate Oral Health and Medicine, US; 4 Harvard Graduate School of Education, US; 5 Oral Health Policy and Epidemiology at HSDM, US

## Abstract

**Purpose:**

The Global Health Starter Kit (GHSK) is an interdisciplinary, competency-based, open access global health curriculum covering global disease and demographic trends, Millennium Development Goals (MDGs) and Sustainable Development Goals (SDGs), the connection between oral health and overall health, social determinants of health, and concepts of sustainable and ethical global health programs. In this study, we evaluate and describe barriers to and facilitators for using and implementing the GHSK curriculum across a variety of new users.

**Methods:**

This two-phase study uses the Roger’s Adoption Curve concept to standardize this evaluation and inform a strategic plan for continuing to move the curriculum across the chasm from early adopters to an early majority of global oral health educators and learners. We utilized a theoretical adoption framework to identify facilitators and barriers under the domains of innovation and curricular, educator and learner, and institutional and structural factors. Under qualitative Phase 1, five early adopter institutions were interviewed to elicit understanding of factors that contribute to adoption of the GHSK curriculum. Common themes identified were next used to create a Phase 2 quantitative survey for early majority subscribers of the GHSK (N = 27).

**Results:**

These qualitative and quantitative results showed an overall high satisfaction with the quality of the GHSK materials, but also effectively identified barriers to its adoption, including inexperience of faculty in teaching global oral health, a lack of awareness and marketing, and absence of global health accrediting requirements.

**Conclusions:**

By identifying the barriers and facilitators of GHSK curriculum integration, this study provides concrete and specific opportunities to improve its format, relevance, content, and delivery. This study outlines next steps to creating a standardized approach to successfully adopting competency-based global oral health teaching and learning.

## Introduction

Oral diseases are the most common chronic conditions worldwide [1]. Dental caries, periodontal disease, and oral cancers effect over 3 billion people globally [2], resulting in suffering from dental pain, days of missed school and work, chronic oral infections, inability to eat and thrive, and overall poor quality of life [3,4]. As a part of systemic health, oral diseases have serious health consequences and pose a significant burden to public health [5]. To best tackle the global burden of oral disease, interdisciplinary care and integrated approaches for prevention are needed. The basis of interdisciplinary care is founded in the education of health care professionals [6,7,8,9].

Education innovations are critical for the advancement of competency-based global health curricula in dentistry [10,11,12,13,14,15]. The Global Oral Health Interest Group of the Consortium of Universities for Global Health (GOHIG-CUGH) established the Global Oral Health Competency Matrix to support improved preparation of dental students to engage in global settings [16]. Moving one step further, the GOHIG-CUGH partnered with the Harvard School of Dental Medicine (HSDM) in 2017 to create a new competency-based global oral health innovation: the Global Health Starter Kit (GHSK). The GHSK aims to provide practical support for educators and learners who are working toward unified competency-based standards and equipping the future generation of dental professionals with “starting” tools to address the tremendous burden of oral diseases—and their consequences—worldwide [17]. In this paper, we describe our approach for analyzing barriers and facilitators to using the GHSK among current early adopter users so the curriculum can “cross the innovation chasm” to an early majority of global oral health educators and learners.

### Innovation Diffusion Overview

**Figure 1:**
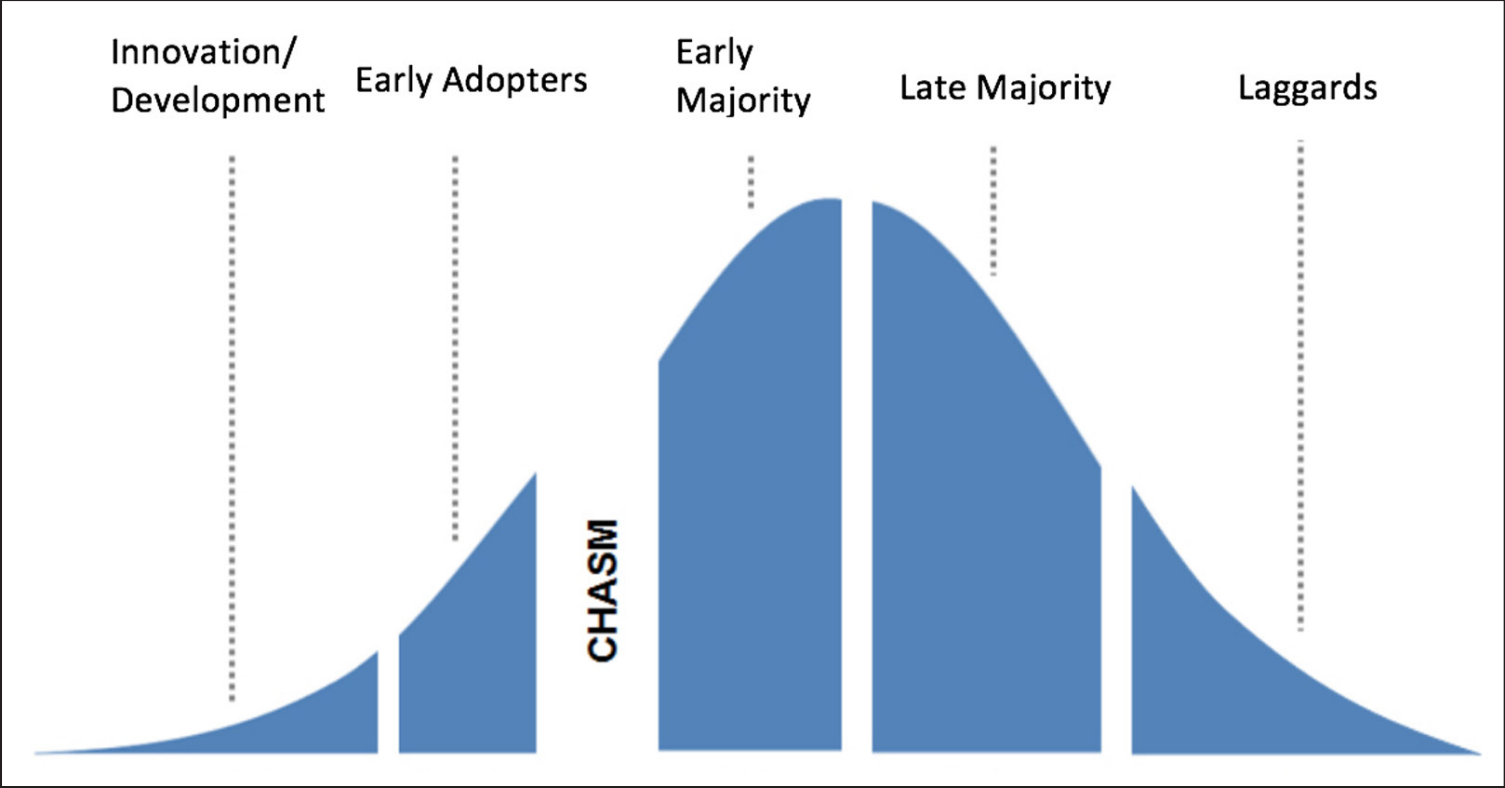
Adoption and Diffusion process for the Global Health Starter Kit curriculum. Adapted from Rogers, E. (2003). The Diffusion of Innovations. Fifth Edition. The Free Press, New York [18].

Rogers defined diffusion as “the process by which an innovation is communicated through certain channels over a period of time among members of a social system” and an innovation was deemed “an idea, practice, or object that is perceived to be new by an individual or other unit of adoption [18].” Innovation diffusion can be analyzed through an adoption curve whose arc rests on the timing of when individual groups within a social system begin using the innovation (***Figure 1***). Five different groups are defined based on distinct attributes that predicate when they will adopt an innovation. These groups are classified as 1) Innovators: venturesome people who bring about change, 2) Early Adopters: respected leaders who try new things, 3) Early Majority: those who adopt new ideas just before the average member of a social system, 4) Late Majority: skeptics who wait until the majority of people are using it, and 5) Laggards, people set in tradition who are the last to adopt new ways [18]. The separation between early adopters and early majority, referred to as “the chasm,” challenges innovators to capture the similarities between the two in order to cross “the chasm” and achieve innovation diffusion [18]. This adoption analysis has been used to show trends of adopting new technologies, even in dentistry, and serves as an ideal example for curriculum innovation adoption as well [19,20].

### The Innovators and Early Adopters

The Global Health Starter Kit (GHSK) serves as the innovation in this paper and was developed by HDSM and GOHIG-CUGH faculty and expert innovators. Initially piloted at the innovator school (HSDM), the purpose of the GHSK is to establish a global health educational foundation by providing a competency-based curriculum that links classroom learning to experiential learning opportunities in the field. The interdisciplinary kit consists of five thematic global oral health modules: 1) Global Trends; 2) Global Goals; 3) Back to Basics—Primary Care; 4) Social Determinants and Risks; and 5) Ethics and Sustainability. Each module is available in two formats, for the teacher or for the learner, and contains measurable learning objectives, teaching presentations and guides, instructional videos, recommended readings, and example assessments. Five early adopter institutions were selected through an application process to work directly with HSDM on adapting and integrating the GHSK into their own global health curricula. These GHSK activities have been described in more detail in previous papers [21,22].

### The Early Majority

Following early adoption at the five initial institutions, the GHSK was launched publicly for open-access use in December 2018. Upon registering to use the kit, subscribers identified themselves as dentists, dental therapists, dental students, public health professionals, dental and medical academicians, and other personnel, including nursing educators and international student medical associates. These individuals represent the first members of the early majority for competency-based global oral health education.

### Adoption

Research on curricular innovations is needed to determine what factors impact the success of its adoption. This study uses an amended version of the Roger’s Adoption Curve concept for diffusion of new technology as it applies to curricular innovation and specifically to the dissemination of the GHSK. By evaluating barriers and facilitators to using and adopting the GHSK, innovators can develop a strategic plan for continuing to move the curriculum across the chasm from early adopters to an early majority, thus increasing access to competency-based global oral health training materials worldwide.

## Methods

The Institutional Review Board (IRB) of the Harvard Faculty of Medicine deemed this study exempt (Protocol: IRB19-0724). Analysis of the GHSK adoption process was carried out in two phases: in-depth qualitative interviews with the five early adopters and quantitative surveys of early majority subscribers who registered on the website. (It should be noted that subscribing was not required in order to use the GHSK, so there may be users who did not provide contact information).

### Phase 1: Qualitative Interviews

**Figure 2:**
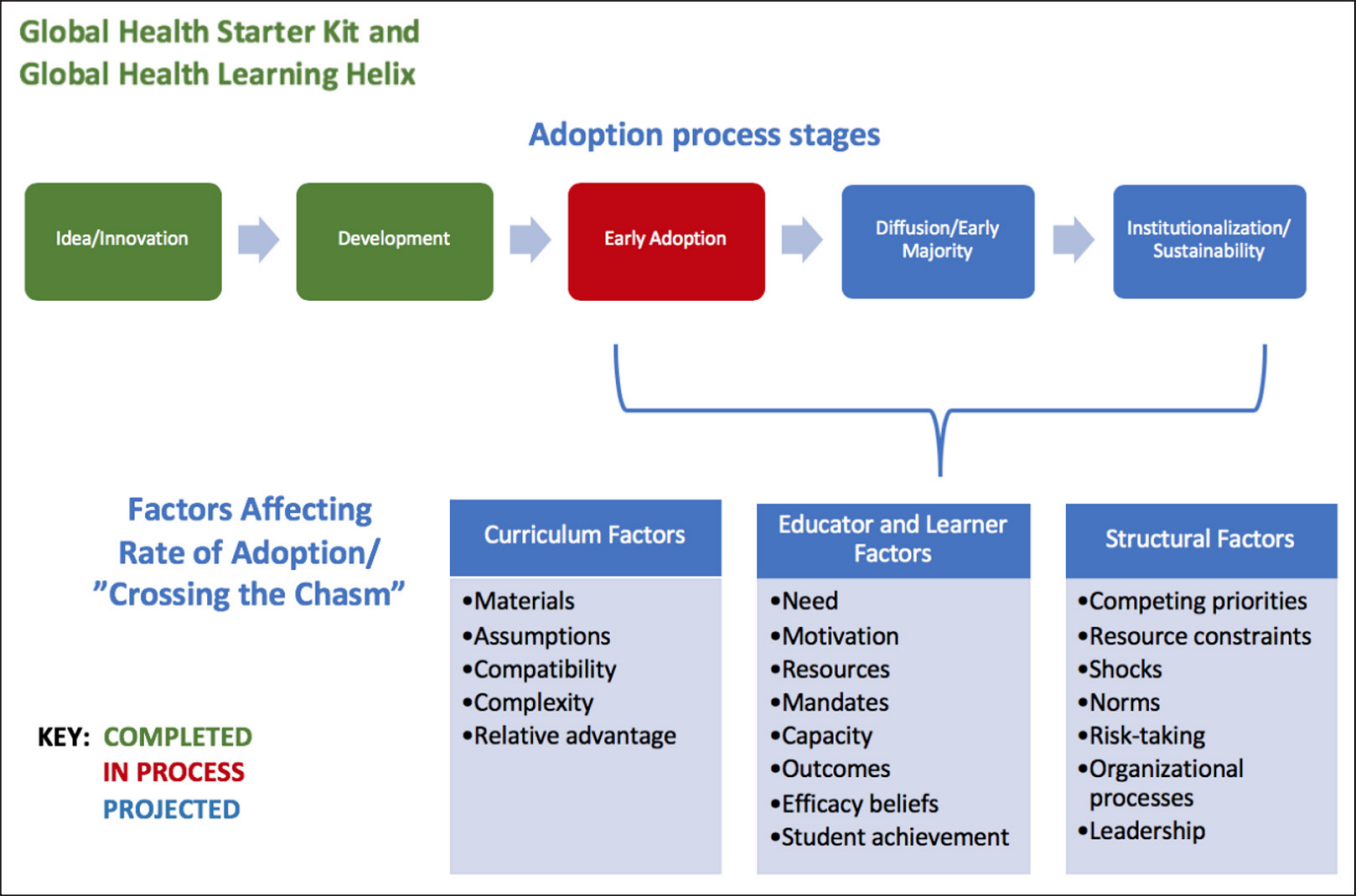
Innovation framework for adoption process and diffusion analysis. Adapted from People and Technology in the Workplace, Figure 1 page 136 [32,33].

A semi-structured interview guide was developed using Roger’s framework of factors affecting the rate of adoption and diffusion. These factors include ‘shocks’ to the current curriculum (i.e., new accreditation standards), motivating and enabling factors of adopter faculty (i.e., resource constraints or faculty interest), innovation factors (i.e., the materials), structural factors (i.e., competing curricular requirements), assumptions and values of the innovation team and early adopters, and breadth versus depth of adoption (across a department versus a standalone class). The factors were constructed into three domains: curriculum and innovation, educator and learning, and institutional and structural (***Figure 2***).

Individuals selected to participate in the interviews were pioneers at each of the five early adopter institutions. Six individuals, one to two interviewees from each institution, were invited to participate through email. The interviews were conducted using video or audio calling and ranged from 25 to 45 minutes. Consent was obtained and a total of five hours of audio was recorded and transcribed with personal identifiers removed.

The deidentified transcribed interviews were aggregated, placed into a single document, and analyzed using the three established domains described above to guide the process. Themes were identified in the transcripts within each domain (***Table 1***). The transcripts were then analyzed to define specific codes or key concepts within each theme. A second revision of the themes and codes was conducted to ensure they reflected the transcribed content. The themes and codes determined in this analysis of early adopter interviews set the foundation for the development of the early majority quantitative survey.

**Table 1:** Early Adopter Interview Qualitative Analysis of codes categorized into themes under three major domains. N = 6.


DOMAIN	THEME	CODE	QUOTES

**Curriculum/ Innovation Factors**	Content (conceptually)	Already complete, some exercises too advance or less connected to our school, higher level than students would appreciate, serves as an intro to public health and global approaches, good quality, meets a need and is addressing a knowledge gap	Great material especially as a training before going to a field project! It really targets specific things, like the SDG’s, oral health and how it connects with general health and universal care

	Materials (physical presentation)	Limited by English, need all open access readings (no paywalls/subscriptions), good use of PowerPoint, videos, and written explanations, interactive, missing take home assignments, takes advantage of technology, good to have video for teacher and other for student, friendly for a teacher, quizzes were instrumental to measure success, needs experiential component, ability to adapt/integrate/merge into existing curriculum	Though the video transcripts were available for help, the course delivery could have been slightly slower especially considering a large number of non-native English speakers who would benefit from the course

	Delivery	Delivery shows passion and how much instructor knows, engaging to watch, great opportunity to share through open access, good platform and position to share with others (website), user friendly/easy to find and access materials	Very useful for us both in terms of content and how the learning activities are structured and organized

**Educator and Learner Factors**	Prior learner experience	Lacked global oral health education, want better public health foundation, no epidemiology background, want more preparation before global service-learning trip, learners currently focused on boards	When students graduate and practice in the field, they are lacking in leadership in global oral health and that is why it is important for the students to have it correctly so that when they graduate as a dentist, they can practice efficiently

	Impact on student learning	Broader perspective, digestible for students, use concepts to improve actual outreach programs, better prepared for global service learning trips, students learned a lot, brings level of discussion higher and depth increases, expanded beyond dental students to broader audience, if students across the globe could take this together it would add discussion and a new peer to peer experience	We have received emails from the students who are excited about this and some of them are now applying to oral internship programs

	Educators	Accepted by faculty, current curative mindset, faculty need to be brought up to speed on these principles/faculty development, lack of global health faculty	This is a great opportunity and makes professors have a good tool to strengthen their way to teach these issues and introduce the part of community work fields

**Institutional and Structural Factors**	Institutional acceptance	Accepted by faculty and dean, approved through curriculum, needs to understand why GHSK is relevant	I talked to the associate dean for curriculum, and she saw that it was a very good idea The leadership needs to understand why this is relevant

	CODA	Can’t alter curriculum during accreditation, not required by CODA	This is essential in the curriculum of the dental schools

	Integration into current curriculum	Convert materials from one platform to another, want to integrate into first year so all students receive this education, using in classroom and field setting, merged into what we teach, spread throughout years for the students, worked with scheduler to change the time of the class	What we are able to do at the moment is to integrate the GHSK into public health curriculum, but I think the GHSK should not only be in dental public health curriculum, it should be in all departments

	Institutional Marketing	Created awareness about merging with Harvard curriculum, good internal promotion	You have to PR your course and make it in social media in such a way that the message gets to the students you are targeting

	Interprofessional usage	Taught to public health students	I believe this is a great opportunity to have both sets, school of public health and school of dentistry, so they can do interprofessional collaboration


### Phase 2: Quantitative Survey

A 12 question Qualtrics survey was developed based on the qualitative results to determine the experience of using the GHSK among early majority subscribers. The survey aimed to identify the facilitators and barriers that contribute to or hinder the adoption of the GHSK curriculum within the three domains.

First, survey validation assessed the time needed to take the survey, as well as clarity and ease of use, and adjustments were made. Five HSDM students and four HSDM faculty members were involved in validating the survey; some had experience using the GHSK while others had no exposure to the GHSK. This mixed group of validators was chosen to ensure that questions could be interpreted by GHSK users with various knowledge backgrounds in global health. Next, survey dissemination was completed via individual email addresses that early majority members provided when they subscribed to the GHSK on the website. A total of 74 subscribers were emailed to participate in this study. Three emails were sent over the course of three weeks. Participation in the survey was fully voluntary and served as consent.

## Results

### Phase 1

The transcribed early adopter qualitative interviews revealed eleven themes (content, materials, delivery, prior learner experience, impact on student learning, educators, institutional acceptance, Commission On Dental Accreditation (CODA), integration into current curriculum, institutional marketing, and interprofessional usage) within the three domains. Each theme had between one and sixteen codes (***Table 1***). Overall, early adopter interviewees agreed that regarding Curriculum/Innovation Factors, the GHSK content was of high quality and was presented in a user-friendly interactive format that was easy to share with others through the online, open-access format. Critiques of the GHSK materials included that they were only available in English, there was limited access to cited journal articles behind paywalls, and there were not enough pre-made quizzes or competency assessments available for testing learner outcomes.

The early adopters also identified barriers related to Educator and Learner Factors, including lack of prior experience in global oral health, student focus on regulatory body exams (i.e., national board exams), and lack of global health faculty. Enabling categories within these factors included students wanting more preparation before global service-learning trips, students reporting they were better prepared for and had broader perspectives during their field experiences, and faculty from every department (not solely public health) advocating for the value of oral global health education.

Institutional and Structural Factors identified by early adopters that could enable curricular adoption included acceptance by faculty and the dean/school leadership, institutional curriculum approval, and student awareness of the GHSK. One interviewee commented that their institution created a marketing strategy for the GHSK. Barriers included a lack of intuitional understanding to the relevance of the GHSK, failure by CODA to require curriculum content, and difficulties scheduling a time for teaching the GHSK.

### Phase 2

The quantitative survey sent to early majority stakeholders received 27 complete responses, earning a response rate of 36.5%. Respondents identified themselves as: student/trainee (33.3%), dental educator (29.7%), other health professions educator (medical, nursing, public health, etc.; 25.9%), or clinical dentist (11.1%). Of the respondents, 51.8% of GHSK subscribers were educators, while 48.2% were learners. Overall, the GHSK material qualities were rated positively, with 100% of respondents either strongly agreeing or somewhat agreeing for each listed material quality. The majority (69.2%) of respondents were extremely satisfied with the GHSK overall, while 30.8% were moderately satisfied. All of the respondents agreed that their users acquired new knowledge/skills, understood the relevance of the GHSK, and that the materials broadened their perspective in the field.

Educator respondents comprised of 7.1% clinical dentists, 50.1% dental educators, 28.5% other health professions educators, and 14.3% student/trainees. Ninety percent of educators indicated that they were using these materials prior to a global field experience (i.e., mission work, research). The materials were being used in a standalone program by 45.5% of respondents and integrated into an existing curriculum/program by 75.0% of respondents with some educators indicating both uses at their institution. 92.7% of respondents reported that leadership/supervisors and their organization supported the GHSK. However, only 30% of respondents reported that there were marketing strategies in place to increase awareness of the GHSK.

**Figure 3:**
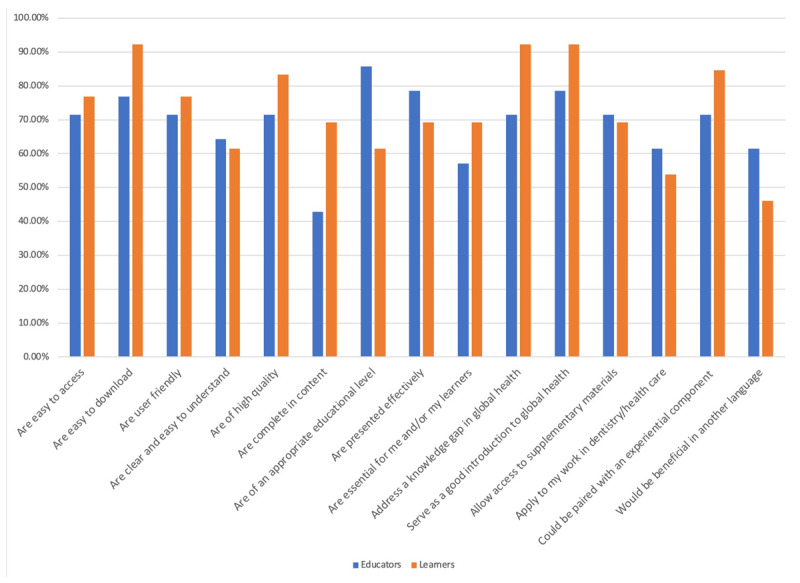
Educator and learner respondents who rated “strongly agree” with each GHSK Curriculum/Innovation Factor. N = 27.

**Figure 4:**
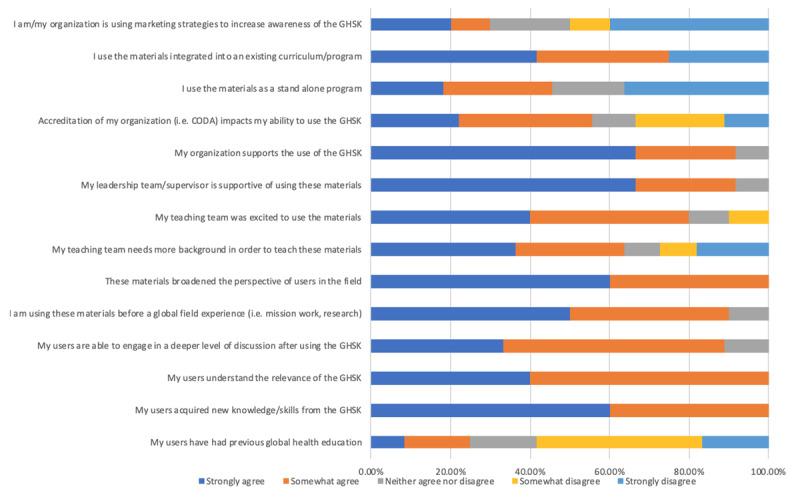
Educator ratings of their level of agreement with specific statements about the Educator and Learner Factors and Institutional and Structural Factors. N = 14.

Learner respondents comprised 15.3% clinical dentists, 7.7% dental educators, 23.1% other health professions educators, and 53.9% student/trainees. Of these learners, 41.7% reported that they had previous global health education, and 100% agreed that they acquired new knowledge/skills from the GHSK. Learners reported that they understood the relevance of the GHSK and were able to engage in deeper discussion after using the GHSK. Seventy-five percent of learner respondents were using the materials prior to a global field experience (i.e., mission work, research) and 83.3% agreed that the materials broadened their perspective during field experiences. See ***Figures 3*** and ***4*** for details.

**Figure 5:**
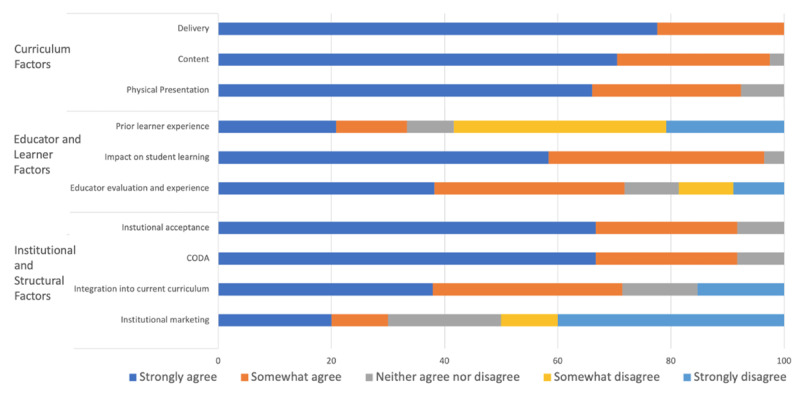
Breakdown of GHSK users’ level of agreement for factors facilitating their use of the GHSK. N = 27.

Curriculum/innovation factors were rated highly, with positive remarks on content, delivery, and physical presentation. Education and learner factors had a more diverse range; learner experience varied among participants, and educators indicated they required more background on the topic in order to teach the materials. Institutional and structural factors had the greatest variation of answers (***Figure 5***).

## Discussion

Previous studies have illustrated slow adoption of emerging innovations in dental education [23,24]. Challenges have included uneasy integration, constraints in the curriculum, lack of standardized materials, and negative perceptions of those who had not yet engaged with the innovation [10,13,25,26]. Through analysis of the process by which the innovator school, the five early adopter institutions, and the first wave of the early majority utilized the GHSK, we can derive data-driven opportunities that are crucial for the GHSK to cross the chasm to early majority use.

Under the innovation domain, our results identified the high quality of the GHSK material content, its physical presentation, and ease of delivery as key factors in facilitating curriculum adoption. Overall, in terms of innovation factors, minimal barriers to adoption were evidenced. This is expected and understandable as innovation is the domain most easily controlled and planned out. However, the GHSK can and should be translated into different languages to further assist in diffusion. Secondly, the addition of premade evaluations could allow for student assessment. Learner self-assessments could serve as a tool for users to gauge their level of understanding and identify areas needed for further study [27,28].

With regard to the learner and educator domain, current literature shows that both trainees and teachers lack background knowledge in global health. A study of US dental school academic affairs deans showed that although global health topics were covered in dental curricula, only 12% of schools had a course dedicated to global oral health [29]. Students desire greater exposure [2,19], but faculty members’ lack of knowledge about specific topics or lack of standardized teaching materials prevent course development [13]. This suggests the need for more supplemental materials to prepare educators for teaching global oral health content. Dental institutions can also call on educators of different disciplinary backgrounds to create an interdisciplinary approach for teaching global oral health [13,30,31]. The GHSK innovation creators can also host faculty development webinars and workshops for those interested in using the materials in their classrooms and programs.

Finally, strategic steps for GHSK adoption must be addressed on institutional and structural levels. Individuals or groups hoping to integrate the GHSK must acquire support from fellow educators and organization leadership (i.e., deans, course directors, etc.). Creating awareness for the curriculum at the institutional level through ready-made marketing materials like shareable videos and summary documents could also increase support. The American Dental Association’s CODA, which accredits dental education programs in the US, does not mandate global health education. Thus, an alternative approach could be to integrate GHSK content into an existing curriculum/program. Indeed, 59% of US dental schools academic affairs deans reported they plan to incorporate global oral health education into their curricula, either through global experiences for their students or didactic content [19]. Perhaps most immediately, to complement the increasing popularity of global service learning in dental education, GOHIG-CUGH can work with educators and leadership to transition away from standalone one-off outreach programs toward normalizing integrated global experiences that are part of a standardized competency-based educational foundation. Resources for educators and learners can be found through attending conferences or webinars on the topic or on the Global Health Starter Kit webpage.

This study is limited by several factors. First, defining who qualified as the “early majority” and quantifying that group was challenging and resulted in a small sample size of respondents, particularly because currently there is no formal measure for global oral health in dental curricula. Thus, any user adopting the GHSK following our early adopter pilot was considered the beginning of the next phase in the innovation curve, the early majority. The small sample size of both the early adopters and early majority could bias the data and may not be representative of all institutions. Second, due to the nature of the quantitative survey format and lack of required registration prior to use of the open-access Starter Kit materials, the initial 35% response rate was low and likely not generalizable to all users. Third, the population of individuals who were included in this study self-identified themselves as being interested in global oral health by subscribing to the GHSK. As a result, responses from this survey may not be representative of all institutions interested in adopting competency-based global oral health curricula, including but not limited to the GHSK. Future data collection should strive to represent adoption institutions more broadly. Nevertheless, our results provide information about the motivators and barriers to curriculum adoption and what interventions may be needed to further aid distribution. As of December 2020, the GHSK has registered subscribers from five continents and thirty-five different countries. Translation to Spanish is also being planned. Thus, we plan to continue to learn from new adopters as registrants grow in number through a long-term analysis of the GHSK adoption.

## Conclusions

This study identified barriers and facilitators affecting the rate of diffusion along an innovation adoption curve of the GHSK in three domains: curricular/innovation, educator and learner, and institutional and structural factors. The GHSK itself was regarded as high quality with comprehensive and appropriate content according to survey respondents. Barriers to adopting the GHSK were primarily found in the latter two domains, and included faculty unprepared to teach global health content, lack of awareness and marketing of the curriculum, and lack of institutional prioritization in already demanding curricular schedules because global health is not explicitly required by an accrediting body. Defining concrete, specific opportunities for improvements in curriculum format, relevance, content, and delivery should assist in further adoption. Strategic partnerships between current and new subscribers could increase diffusion by establishing purposeful communication channels, a user community of practice, and promising practices for curricular integration. Identifying areas of improvement in adoption and diffusion processes could enable strategic steps to “crossing the chasm,” moving from early adopters to the early majority and achieving integration of competency-based global health teaching and learning in dental education. Ultimately, the results of this project could have implications for health professions and global health education more broadly through a potentially generalizable model of curricular evaluation, adoption, and diffusion strategies.
